# Predictive and Prognostic Biomarkers of Recurrence in Locoregional Colorectal Cancer

**DOI:** 10.7150/jca.111828

**Published:** 2025-07-01

**Authors:** Alicia Sánchez Cendra, Leonel Pekarek, Linda Rocio Ospino, Yumna Dbouk, Sami Chnaiker, Ana Luengo, Tania Villamor, Amalia Corralo, Beatriz Pedrejon, Tatiana Pekarek, Silvestra Barrena-Blázquez, David Díaz-Pérez, Ana M Minaya-Bravo, Laura López-Gonzalez, Manuel Díez Alonso, Tomás Ratia Giménez, Alberto Gutiérrez-Calvo, Melchor Álvarez-Mon, Javier Cassinello Espinosa, Raúl Díaz-Pedrero, Miguel A Ortega

**Affiliations:** 1 Oncology Service, Guadalajara University Hospital, 19002 Guadalajara, Spain.; 2 Department of Surgery, Medical and Social Sciences, Faculty of Medicine and Health Sciences, University of Alcala, 28801 Alcala de Henares, Spain.; 3 Ramón y Cajal Institute of Sanitary Research (IRYCIS), 28034 Madrid, Spain.; 4 Department of General and Digestive Surgery. General and Digestive Surgery, Hospital Universitario Príncipe de Asturias, 28805 Alcala de Henares, Madrid, Spain.; 5 Deparment of Medicine and Medical Specialities (CIBEREHD), Faculty of Medicine and Health Sciences, University of Alcalá, 28801 Alcala de Henares, Spain.; 6 Department of General and Digestive Surgery, General and Digestive Surgery, Hospital Universitario de Torrejón, 28850 Torrejón de Ardoz, Madrid, Spain.; 7 Inmune System Diseases-Rheumatology and Internal medicine Service, Hospital Univer-sitario Príncipe de Asturias (CIBEREHD), 28806, Alcala de Henares, Spain.

**Keywords:** Serological biomarkers, colorectal cancer, histological markers, genetic biomarkers, microRNA, circulating tumor cells.

## Abstract

Colorectal cancer (CRC) remains a major global health challenge, as recurrence and metastasis continue to significantly impact patient survival and management, especially in cases of locoregional disease. In this regard, identifying reliable predictive and prognostic biomarkers is essential not only for improving therapeutic strategies but also for personalizing treatment plans and guiding post-treatment surveillance. This article comprehensively reviews histological, genetic, microRNA (miRNA), circulating tumor cell (CTC), and serological biomarkers, all of which are closely linked to recurrence and metastatic progression in locoregional CRC.On the one hand, histological markers, such as tumor budding and lymphovascular invasion, offer crucial prognostic insights regarding disease aggressiveness. On the other hand, genetic alterations, including mutations in KRAS, BRAF, and TP53 genes, serve as predictive indicators for therapeutic response as well as risk of recurrence. Moreover, specific miRNAs, such as miR-21, have emerged not only as diagnostic but also as prognostic tools due to their association with metastasis and chemoresistance. Furthermore, circulating tumor cells and cell-free DNA (cfDNA) released by tumors into the bloodstream represent non-invasive biomarkers that are useful for the early detection of micrometastatic disease and real-time monitoring of therapeutic efficacy. Additionally, serological markers, including carcinoembryonic antigen (CEA) and carbohydrate antigen 19-9 (CA 19-9), continue to play key roles in the routine surveillance of CRC. Integrating these biomarkers into clinical practice thus holds significant potential to stratify patients at high risk of recurrence and guide personalized therapeutic approaches, ultimately improving outcomes for CRC patients. This review consolidates recent findings and underscores the ongoing need for further studies to validate these biomarkers across larger patient cohorts and various CRC stages.

## Introduction

Colorectal cancer (CRC) is a pressing global health issue, ranking as the third most commonly diagnosed malignancy and the second leading cause of cancer-related deaths worldwide, following lung cancer. In 2020 alone, approximately 1.9 million people were diagnosed with CRC, and nearly 935,000 lives were lost to this disease 1,2. These staggering numbers highlight the significant burden of CRC and the urgent need for effective prevention, early detection, and tailored treatment strategies.

CRC incidence shows notable geographic differences, with the highest rates reported in developed regions such as North America, Europe, and Oceania. This disparity is often linked to lifestyle and environmental factors typical of high-income nations, including diets rich in red and processed meats, sedentary behavior, obesity, smoking, and alcohol consumption 3,4. Meanwhile, regions like Africa and South-Central Asia exhibit comparatively lower CRC rates, likely due to differences in diet and lifestyle. However, as these areas increasingly adopt "Westernized" habits, the incidence of CRC is expected to rise significantly, with projections suggesting over 3 million new cases annually by 2040—a growth of more than 60% compared to current levels 5.

Even within high-incidence countries, disparities persist across socioeconomic groups. Lower-income populations often experience higher CRC rates, likely due to reduced access to healthcare, fewer opportunities for screening, and delayed diagnoses 6. In contrast, nations with well-established screening programs, such as the United States and many European countries, have seen improvements in early detection and survival rates. Yet, CRC continues to account for nearly 10% of all cancer-related deaths globally, emphasizing the need for consistent progress in addressing this disease 7,8.

One of the biggest challenges in managing CRC is the high risk of recurrence, particularly for patients with locoregional disease. Despite advances in surgery and adjuvant therapies, recurrence remains a complex and multifactorial issue. It can manifest either as local recurrence, near the primary tumor site, or as distant metastases, most commonly in the liver and lungs. Studies suggest that 30-50% of patients with locoregional CRC will experience recurrence within the first five years after treatment, even if curative resection was achieved 9.

Recurrence risk is closely tied to several clinical and pathological factors. Disease stage at diagnosis is one of the most critical predictors; patients with stage III CRC, where lymph node involvement is present, face a significantly higher recurrence risk compared to those with stage II disease. Nearly half of stage III patients experience some form of recurrence, underscoring the importance of rigorous post-treatment monitoring 10. Additional histopathological markers—such as lymphovascular invasion, perineural invasion, and tumor budding are also strongly linked to recurrence. For example, lymphovascular invasion, which reflects the spread of cancer cells into blood or lymphatic vessels, is associated with both local and distant metastases 11,12.

The quality of surgical resection also plays a pivotal role. Positive surgical margins, which indicate the presence of cancer cells at the edges of resected tissue, are associated with a markedly higher risk of locoregional recurrence. Conversely, achieving negative margins, where no cancer cells are detected, significantly reduces recurrence risk and improves prognosis. These findings highlight the critical importance of adhering to rigorous oncological surgical standards 13.

Beyond these clinical factors, genetic alterations are central to understanding CRC recurrence. Mutations in oncogenes and tumor suppressor genes such as KRAS, BRAF, and TP53 not only drive tumor progression but also influence recurrence. For instance, KRAS mutations are linked to resistance to anti-EGFR therapies, limiting treatment options and increasing the likelihood of recurrence. Similarly, the aggressive BRAF V600E mutation has been associated with poorer outcomes and higher recurrence rates, emphasizing the value of genetic profiling to identify high-risk patients 14,15.

Given the high rates of recurrence, recent research has focused on developing better follow-up strategies and predictive biomarkers. Circulating tumor cells (CTCs) and cell-free DNA (cfDNA) have emerged as valuable tools for detecting micrometastatic disease and monitoring recurrence in real time. These biomarkers provide a non-invasive method to identify early molecular changes that signal recurrence, enabling timely interventions even before clinical symptoms appear 16.

## Serological Biomarkers of Recurrence

Serological biomarkers are valuable tools for the diagnosis, monitoring, and prognosis of colorectal cancer (CRC), although they have inherent limitations in specificity and sensitivity 17. Among the primary biomarkers routinely used in clinical practice for CRC diagnosis and response monitoring are carcinoembryonic antigen (CEA), carbohydrate antigen 19-9 (CA 19-9), and CA 125. Additionally, Tissue Polypeptide Specific Antigen (TPS) and tumor-associated glycoprotein 72 (TAG-72) are also considered useful 18. It is important to note that certain conditions, such as diabetes, as well as liver, pancreatic, and ovarian diseases, can lead to elevated levels of CEA, CA 19-9, and CA 125. If these influencing factors are not accounted for in clinical practice, there is a risk of obtaining false-positive results during the evaluation of CRC patients 19. The biomarkers described below are summarized in Table 1.

Carcinoembryonic antigen (CEA) is a glycoprotein expressed in epithelial cells of the gastrointestinal tract, playing roles in biological processes such as cell adhesion and apoptosis 20. This protein is a well-studied tumor marker widely used in CRC management, particularly for surveillance following curative surgery and for detecting metastases. Studies indicate that a rapid decrease in postoperative CEA levels correlates with a better prognosis. In contrast, elevated CEA levels are associated with an increased risk of recurrence or progression and are considered an independent predictor of survival in patients with elevated preoperative CEA levels 21.

The presence of CEA in the bloodstream plays a critical role in metastasis, particularly to the liver, as CEA facilitates the adhesion of tumor cells to hepatic tissues through interactions with specific receptors. Experimental studies have demonstrated that suppressing the CEA receptor (CEAR) in CRC cells reduces their invasive potential and tumor progression. These findings underscore the importance of CEA in the metastatic process and its potential as a therapeutic target 22. After surgery, CEA levels are periodically measured to monitor for recurrences. A sustained increase in CEA levels may serve as an early indicator of recurrence, enabling interventions before the onset of clinical symptoms. A multicenter retrospective study on carcinoembryonic antigen (CEA) monitoring in post-surgical colorectal cancer patients included 1,832 stage II and III cancer patients, of whom 1,008 had complete follow-up data. This study revealed that elevated postoperative CEA levels were significantly associated with worse outcomes, including higher recurrence rates and reduced overall and progression-free survival. Patients with high postoperative CEA levels demonstrated lower survival rates, with CEA serving as an independent prognostic factor for progression and survival in this group. Furthermore, increases in CEA levels both before and after surgery correlated with more advanced pathological features, such as lymphatic and vascular invasion, underscoring the value of this marker in postoperative monitoring 23. While an ideal biomarker would exhibit high sensitivity for the early detection of colorectal cancer, CEA does not meet these criteria. Its sensitivity for detecting early-stage CRC is limited, and its use for this purpose is not recommended.

Carbohydrate antigen 19-9 (CA 19-9) is more commonly used in diagnosing pancreatic cancer, with its sensitivity being lower than that of CEA for colorectal cancer detection. Elevated preoperative CA 19-9 levels may correlate with an unfavorable prognosis in CRC. In a study involving 495 patients, CA 19-9 was found to have prognostic significance independent of Dukes staging and CEA levels, highlighting its potential as an additional prognostic factor 24. However, in the follow-up of colorectal cancer patients, CA 19-9 is less informative than CEA. Studies on recurrence have shown abnormalities in CEA in 84% of cases, compared to 48% with CA 19-9, indicating that CA 19-9 is less sensitive for early recurrence detection 25.

Tissue Polypeptide Specific Antigen (TPS) is a biomarker derived from cytokeratin 18 fragments and is associated with tumor cell proliferation. It is generated during various stages of the cell cycle (S or G2) and is released into the tissue following mitotic division. Consequently, the concentration of TPS in the blood, which closely reflects the proliferation rate of cancer cells, serves as an indicator of tumor cell division 26. Elevated TPS levels in CRC patients are observed in approximately 60-80% of cases and are associated with reduced survival, potentially indicating advanced disease or metastases 27.

Tumor-Associated Glycoprotein 72 (TAG-72) is a molecule produced in endothelial cells of the bile ducts, stomach epithelium, and renal pelvis cells 28. It is advisable to evaluate TAG-72 in combination with other markers, particularly CEA. Guadagni *et al.* found that at least one tumor biomarker (CEA, TAG-72, or CA 19-9) was elevated in approximately 61% of colorectal cancer patients 29.

Following the analysis of these biomarkers, a retrospective study involving 102 patients with metastatic colorectal cancer (mCRC) treated with anti-EGFR monoclonal antibodies combined with first-line chemotherapy examined the relationship between baseline levels of TPS, CEA, and CA 19-9 and clinical outcomes, such as progression-free survival. Interestingly, no significant association was found between initial TPS levels and treatment outcomes, while other markers, such as CA 19-9, demonstrated prognostic relevance 30.

Finally, the continuous pursuit of novel tools for colorectal cancer monitoring, follow-up, and prognosis remains a priority. Among these, prospective studies are validating the use of circulating progastrin (hPG80). This precursor of the gastrin hormone has been identified in the blood of patients with various cancers, including colorectal cancer. Its presence in plasma is associated with tumor progression, and it may serve as a biomarker for early detection and disease monitoring, as variations in hPG80 levels correlate with treatment efficacy. Studies have shown that hPG80 is detectable across different stages of colorectal cancer, from early to metastatic disease 31.

Li *et al.* analyzed trajectories of tumor markers, including CEA, CA 19-9, and CA 125, over three years following surgery and their influence on colorectal cancer (CRC) outcomes. They identified three patterns: stable low levels, early increase, and late increase. Patients in the early and late increase groups exhibited a higher risk of recurrence and mortality compared to the stable group, with significant correlations in overall survival (OS) and recurrence-free survival (RFS), independent of other prognostic factors. Additionally, the joint trajectory group emerged as the most significant variable for predicting survival outcomes. Patients with one or more elevated markers had poorer prognoses, and those with rising CA 19-9 and CA 125 levels were categorized as high-risk. This study represents the first longitudinal investigation into the relationships between multiple tumor markers and clinical outcomes in CRC 32.

In conclusion, serological biomarkers play a critical role in the management of colorectal cancer (CRC), providing valuable insights for diagnosis, monitoring, and prognosis. Among the most widely used markers, CEA remains essential for postoperative surveillance and early recurrence detection, while CA 19-9 offers additional prognostic value despite its lower sensitivity compared to CEA. Emerging biomarkers, such as hPG80, show promising potential for early detection and treatment monitoring, highlighting the evolving landscape of CRC biomarker research. However, the utility of these markers is often influenced by external factors, such as comorbid conditions, and further longitudinal studies are needed to refine their predictive accuracy. The integration of multiple markers, as demonstrated in recent research, may offer a more robust approach to predicting clinical outcomes and guiding therapeutic decisions.

## Genetic and Histological Risk Markers

Both histological biomarkers, such as tumor differentiation grade and lymph node invasion, and genetic biomarkers, including KRAS and BRAF mutations as well as microsatellite instability (MSI), are essential in colorectal cancer (CRC) for identifying patients at higher risk of recurrence or progression. Techniques such as immunohistochemistry and polymerase chain reaction (PCR) are commonly employed to analyze these markers. The study of these biomarkers helps optimize treatment strategies and improve clinical outcomes for CRC patients. The most relevant markers and their utilities are summarized in Table 2.

Approximately 70% of CRC cases arise sporadically 33, although a significant subset presents a notable genetic predisposition. In CRC, detecting genetic mutations in tumor histological samples is crucial for tumor characterization and classification, as this information provides insights into the cancer's biology, aiding in the prediction of treatment responses and patient prognosis.

According to Marisa, L., *et al.*, CRC develops through multiple pathways influenced by diverse molecular characteristics 34. Currently, the development of CRC (both sporadic and hereditary) is thought to occur through at least three primary pathways: microsatellite instability, mismatch repair deficiency, and the CpG island methylator phenotype (CIMP, involving epigenetic changes). Each pathway leads to neoplasms with distinct genotypes and phenotypes 35.

Regarding the development of CRC, Shen, L., *et al.* 36 identified two potential triggering events. The first suggests that a series of genetic events may activate methyltransferases or inactivate factors that protect against methylation, impacting several tumor suppressor genes, including hMLH1, p16, and p14. The second proposes that CRC etiology is linked to environmental factors, where methylation could result from the interaction between environmental exposures and age-related genetic changes. This interaction may be associated with events such as chronic inflammation or exaggerated responses to tissue injury, as chronic inflammation has been correlated with elevated methylation levels 36.

Pathological staging is a widely used method to assess recurrence risk in CRC patients after surgery and to determine the need for adjuvant chemotherapy based on tumor stage. However, evaluating the tumor's genetic burden may provide critical prognostic insights and facilitate the selection of targeted therapies. This, in turn, could improve overall survival rates 37.

Histological and genetic biomarkers are fundamental tools for identifying recurrence risk and tailoring treatments in colorectal cancer (CRC). Key markers such as KRAS, BRAF, MSI, and tumor differentiation offer valuable insights into tumor behavior and prognosis. Incorporating these biomarkers into clinical practice enables more personalized therapeutic approaches, improving patient outcomes. The next section will delve into these biomarkers, exploring their role in CRC progression, prognosis, and therapeutic potential and are summarized in Table 3.

### Pathways Involved in the Development of Colorectal Carcinoma

#### APC Gene

**Figure 1:**
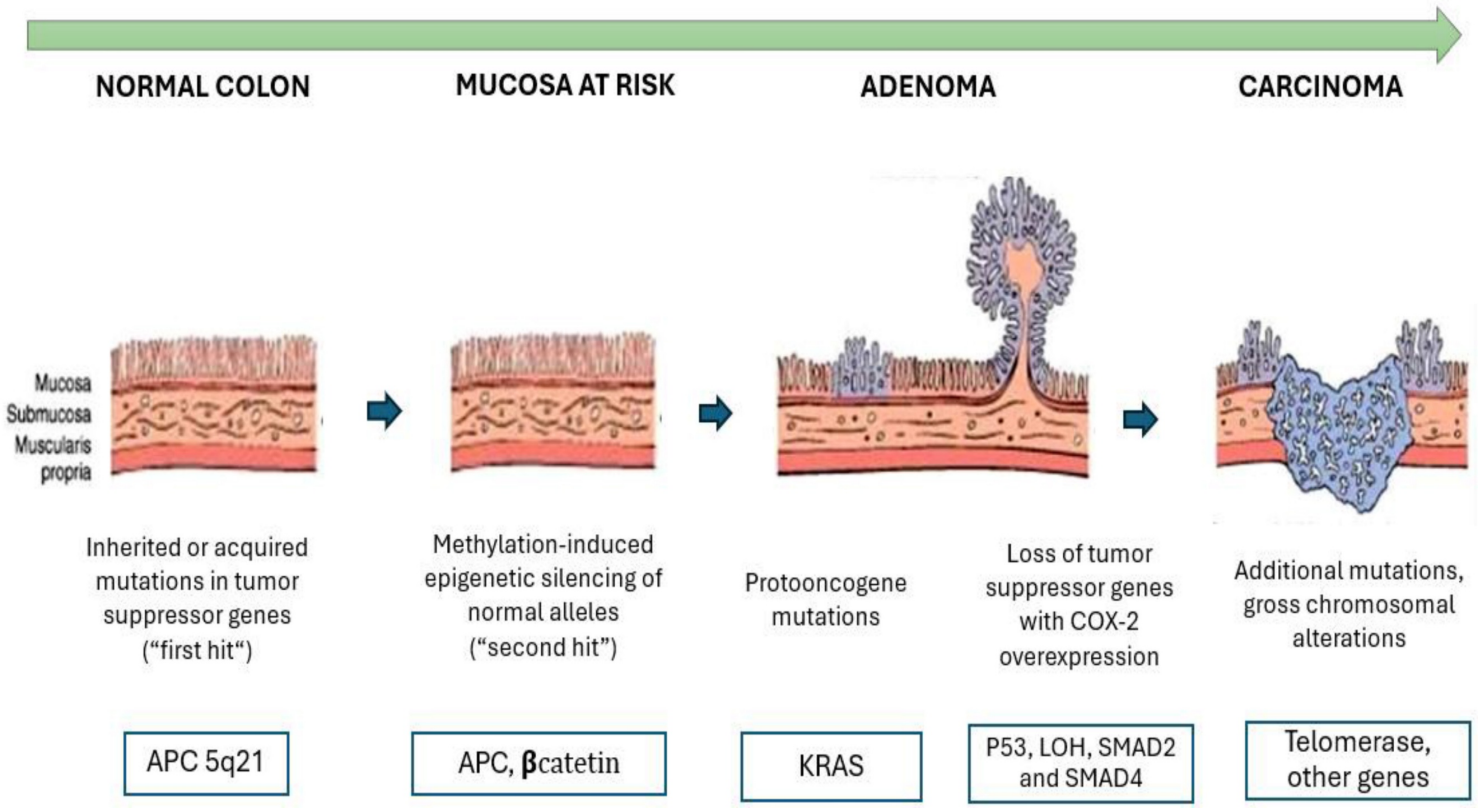
** Colorectal Cancer Progression: From Normal Mucosa to Invasive Carcinoma.** This image illustrates the progression of colorectal cancer through the adenoma-carcinoma sequence. It begins with normal mucosa undergoing mutations in tumor suppressor genes, such as APC (5q21) (first hit), followed by epigenetic alterations and silencing of normal alleles (second hit). Over time, proto-oncogene mutations, like K-RAS (12p12), promote uncontrolled growth and the formation of adenomas. If additional mutations accumulate, such as the loss of p53 (17p13) and SMAD2/4 (18q21), along with COX-2 overexpression, the adenoma progresses to an invasive carcinoma with chromosomal alterations and telomerase activation.

The APC gene, located on chromosome 5q, is one of the key genes involved in the development of colorectal cancer (CRC). It plays a significant role in both familial adenomatous polyposis and most cases of sporadic CRC. Its primary function is to encode a multifunctional protein that plays a critical role in the WNT signaling pathway. This pathway is altered in more than 90% of CRC cases. The APC gene plays a key role in the adenoma-carcinoma sequence hypothesis, which explains the progression of colorectal cancer. Initially, normal colonic mucosa undergoes mutations in tumor suppressor genes, such as APC (5q21) (first hit). Subsequently, epigenetic alterations and silencing of normal alleles occur (second hit). The progressive accumulation of these mutations and epigenetic modifications drives the transformation of a normal cell into an invasive carcinoma. This hypothesis is shown in Figure 1. Mutations in this pathway lead to increased levels of beta-catenin in the plasma, which in turn stimulates the expression of oncogenes such as c-myc and cyclin D1 38.

A study by Liang J., *et al.* demonstrated that the I1307K polymorphism of the APC gene is associated with a significantly higher risk of developing CRC, with an odds ratio of 2.17 39. Additionally, Chen TH., *et al.* 40 identified that metastatic CRC patients with APC mutations and elevated miR-21 levels have worse survival rates.

The study of APC mutations holds great potential as a prognostic biomarker; however, its application remains under active investigation.

#### Microsatellite Instability (MSI)

Microsatellite instability (MSI) is a phenomenon associated with deficiencies in the DNA mismatch repair (MMR) mechanism, resulting from the inactivation of key MMR genes such as MSH2, MLH1, MSH6, and PMS2. This deficiency leads to the clonal replication of altered nucleotide sequences, manifesting as DNA deletions or insertions. MSI can be detected using peripheral blood DNA analysis or immunohistochemical techniques on tumor tissue. Among these genes, MLH1 is the most commonly altered.

MSI is observed in both Lynch syndrome and sporadic CRC cases. CRC tumors with MSI exhibit distinct characteristics that set them apart from other forms of CRC. These tumors are predominantly located in the proximal colon, especially near the splenic flexure. They are also more common in elderly patients, with a higher prevalence among women within this demographic. Histologically, MSI-positive tumors often contain mucin and are characterized by poor differentiation.

One of the most notable features of MSI-positive neoplasms is the extensive lymphocytic infiltration, which suggests an active immune response that may benefit patient prognosis. This lymphocytic infiltration has been associated with favorable outcomes, as it reflects a potential interaction between the immune system and the tumor 41.

In prognostic terms, MSI-positive tumors are generally associated with better outcomes at all disease stages compared to MSI-negative tumors 37,42. However, it is noteworthy that these tumors may exhibit resistance to monotherapy with fluoropyrimidine-based chemotherapy 43.

The evaluation of the BRAF gene is particularly relevant in the context of MSI. A study by Wright, M., *et al.* highlights that tumors exhibiting both MSI and BRAF mutations have a poorer prognosis. This is due to the high-risk characteristics frequently observed in tumors with BRAF mutations 44.

Given the significant improvements in prognosis and survival associated with MSI detection, its determination has become a standardized component of molecular studies in CRC. This standardization enables the precise identification of patients who may benefit from specific treatments and personalized therapeutic approaches, thereby enhancing clinical care.

#### KRAS/NRAS Mutations

The **KRAS gene** is a key component of the MAPK signaling pathway, playing a critical role in cellular processes such as proliferation, survival, and differentiation. Mutations in KRAS occur in approximately 45% of patients with metastatic colorectal cancer (mCRC), making it one of the most frequently mutated oncogenes in this cancer type. Similarly, the **NRAS gene**, which encodes a related Ras family protein, regulates similar signal transduction processes. Mutations in both genes disrupt the signaling pathways targeted by epidermal growth factor receptor (EGFR) inhibitors, allowing tumors to continue growing despite therapy.

In KRAS, approximately 90% of mutations occur at codon 12 (e.g., G12D, G12V, G12C), with codon 13 mutations (e.g., G13D) being less common. These genetic alterations are predictive markers of response to EGFR inhibitors such as cetuximab and panitumumab. Mutations in KRAS, particularly at these codons, confer resistance to EGFR-targeted therapies 45.

The CodeBreaK 300 study investigated the efficacy of sotorasib, a KRAS-specific inhibitor, in combination with panitumumab in patients with mCRC harboring the KRAS G12C mutation. This phase III clinical trial demonstrated that the combination significantly improved progression-free survival (5.6 months) compared to standard treatment (2.2 months). These findings highlight the potential of targeted therapies against specific KRAS mutations to improve outcomes for mCRC patients 46.

In the study by Yamashita S., *et al.*, KRAS and NRAS mutations were associated with R1 resections of liver metastases, leading to higher recurrence rates and worse prognosis 47. This underscores the importance of evaluating KRAS mutations to predict response to targeted treatments and the progression of metastatic disease in CRC patients.

Therefore, KRAS/NRAS mutations not only influence prognosis but also play a critical role in therapeutic decision-making, allowing for more effective and tailored treatment approaches.

#### BRAF

The BRAF gene, part of the ERK-MAPK signaling pathway, requires prior activation of RAS for its expression. Mutations in BRAF are present in 10-15% of colorectal cancers (CRC), with BRAF V600E being the most common variant. This mutation is associated with the development of high-grade tumors, which predominantly occur in the right colon and are more frequently observed in women and elderly patients. Furthermore, tumors with BRAF mutations exhibit a high rate of metastatic spread, particularly to the peritoneum and lymph nodes, resulting in a poorer prognosis. Tran B., *et al.*, emphasize that patients with BRAF mutations require closer monitoring due to the high likelihood of metastasis 48. Similarly, Heuvelings DJI., *et al.*, highlight the tendency of BRAF-mutated tumors to develop metachronous peritoneal dissemination in previously treated patients, suggesting the need for more rigorous follow-up with imaging studies compared to patients without this mutation 49.

Regarding treatment options, BRAF mutations are associated with reduced efficacy of EGFR inhibitor therapies. BRAF inhibitors, such as encorafenib and vemurafenib, have been investigated in combination with anti-EGFR agents to improve outcomes in metastatic CRC patients with BRAF mutations 50. Studies have shown that this combination can provide significant benefits in tumor response and survival. Notably, the BEACON CRC study demonstrated that the combination of encorafenib and cetuximab improved overall survival, objective response rate, and progression-free survival in previously treated metastatic CRC patients with BRAF V600E mutations compared to standard chemotherapy 51.

#### PI3K

Mutations in the PIK3CA gene are identified in approximately 20% of CRC cases. These mutations often coexist with KRAS mutations and loss of MGMT gene expression. In tumors with wild-type BRAF, PIK3CA mutations are associated with a poor prognosis, characterized by significantly reduced survival rates. The simultaneous presence of mutations in PIK3CA exons 9 and 20 is also linked to adverse outcomes 52.

A study by Liao X., *et al.*, concluded that regular aspirin use in patients with PIK3CA mutations may be associated with increased survival 53. This finding suggests that adjuvant therapy could play a pivotal role in the management of these patients. Currently, targeted therapies aimed at the PI3K pathway are under investigation, potentially offering new treatment options for patients with these mutations.

#### PTEN

The PTEN gene (phosphatase and tensin homolog) is a critical tumor suppressor gene whose role in colorectal cancer (CRC) has been extensively studied due to its impact on tumor progression and treatment response. Loss or inactivation of PTEN has been associated with increased metastatic potential, which worsens the prognosis in CRC patients 54.

Furthermore, mutations in PTEN, along with PIK3CA mutations, are linked to reduced responsiveness to immunotherapy, particularly EGFR inhibitors 55. This resistance is partly attributed to disruptions in the PI3K/AKT/mTOR pathway, where PTEN plays a fundamental role. Alterations in this pathway interfere with the mechanisms of action of treatments that rely on its integrity to elicit antitumor responses. The presence of mutations in PTEN or related genes underscores the need for personalized therapeutic strategies that account for tumor genetics to optimize clinical outcomes in CRC management.

Interestingly, in rectal cancer, some studies suggest that certain genetic variants of PTEN may differently influence treatment responses. For example, the heterozygous PTEN rs12569998 variant has been observed to increase tumor sensitivity to both radiotherapy (RT) and oxaliplatin-based chemotherapy (CT), leading to improved disease outcomes and survival 56.

The significance of PTEN in cancer extends beyond its role in tumor dissemination. Variations in this gene and its interaction with other genes and treatments can significantly alter patient prognosis, highlighting the need for further research into its potential as a therapeutic target.

#### TP53

The TP53 gene, which encodes the p53 protein, is one of the most extensively studied tumor suppressor genes and holds significant relevance in cancer biology. The p53 protein, often referred to as the "guardian of the genome," plays a critical role in maintaining chromosomal stability and inducing apoptosis in cells with replication errors that cannot be repaired. This function is crucial in preventing the accumulation of mutations that could lead to malignant transformation. Mutations in TP53 are observed in up to 75% of colorectal cancer (CRC) cases, underscoring its role in both tumor initiation and disease progression 57, 58.

Inactivation of p53 through mutations allows tumor cells to evade apoptosis and promotes genetic instability, facilitating the accumulation of additional mutations that drive carcinogenesis. Beyond contributing to tumor formation and growth, TP53 mutations can also affect treatment responses, particularly chemotherapy and therapies reliant on the activation of apoptotic pathways. Current therapeutic strategies aim to restore p53 function or reactivate its disrupted pathways, representing innovative approaches in the treatment of CRC and other cancers 59.

Recent studies, such as the one conducted by Lahoz S., *et al.*, have identified that the co-occurrence of TP53 mutations with mutations in the SMAD4 gene is associated with poorer prognosis in CRC patients 60. This combination of mutations correlates with reduced responsiveness to first-line chemotherapy, highlighting the pivotal role of TP53 not only in tumorigenesis but also in determining prognosis and the sensitivity of CRC to various treatments.

#### NDST4

NDST4 (N-deacetylase/N-sulfotransferase 4) is a tumor suppressor gene located on chromosome 4q26. According to Sheng Tai Tzeng *et al.*, loss of NDST4 in colorectal cancer (CRC) is associated with poorer prognosis due to its role in tumor progression. This finding positions NDST4 as a potential prognostic marker in CRC, suggesting that its presence or expression levels could be utilized to predict disease outcomes and guide treatment decisions for these patients 61.

### Methylation Markers

Aberrant CpG site methylation in cancer cells contributes to the transcriptional silencing of tumor suppressor and DNA repair genes, a phenomenon commonly observed in various cancers, including CRC. In CRC, a significant interplay exists between methylation pathways and high microsatellite instability (MSI-H). Some studies indicate that MLH1 gene silencing in sporadic cancers may result from promoter hypermethylation, contributing to genomic instability 62.

In the context of CRC, tumors can be classified into two phenotypes based on CpG island methylation: high CpG island methylator phenotype (CIMP-high) and low CpG island methylator phenotype (CIMP-low). CIMP-high tumors are more frequently observed in older individuals, women, and in the proximal colon. It is estimated that approximately 20% of sporadic CRC cases exhibit both BRAF mutations and MLH1 methylation, a pattern also seen in sessile serrated adenomas, which are considered precursor lesions in CRC development 63.

The FDA has approved two methylation biomarkers for the detection of colorectal cancer (CRC): SEPT9 and the combination of NDRG4 and BMP3 64. These biomarkers enable early CRC detection, enhancing diagnostic and treatment options at initial stages.

The literature on methylation markers in CRC includes a wide range of biomarkers. For example, Nilsson TK *et al.* reported that methylation of the p14ARF, RASSF1A, and APC genes is associated with poor prognosis, regardless of tumor stage. In contrast, methylation of the O6-MGMT gene might have a protective effect, suggesting that methylation patterns can influence disease progression in various ways 65.

Other studies have identified additional biomarkers. Hur K *et al.* highlighted that hypomethylation of LINE-1 is linked to poor prognosis in CRC patients 66. Additionally, Coppedè F *et al.* found that methylation of genes such as TFAP2E, SPARC, and UGT1A1 contributes to chemotherapy resistance, particularly to drugs like 5-fluorouracil and irinotecan 37. This therapeutic resistance represents a significant challenge in CRC treatment, emphasizing the need for continued exploration of novel therapeutic strategies to counteract these mechanisms of evasion.

Epigenetic regulation in CRC, specifically through methylation, plays a critical role in controlling tumor suppressor genes like CDX2. This gene modulates the Wnt/β-catenin signaling pathway and is involved in the differentiation of intestinal epithelial cells, with expression primarily in the ileum and proximal colon 67.

A critical feature in CRC is CpG island hypermethylation in the promoter regions of genes like CDX2. This hypermethylation can lead to transcriptional silencing of CDX2, reducing its expression and eliminating its protective role in tumor growth control. Studies, such as those by Ilie-Petrov *et al.*, have shown that high levels of CDX2 expression are associated with better survival rates and lower disease recurrence in CRC patients. Conversely, reduced expression of CDX2, mediated by promoter hypermethylation, has been linked to a higher risk of CRC development and progression, as the silencing of this gene facilitates tumor growth. These findings suggest that epigenetic changes in CDX2 could serve as prognostic biomarkers and potentially as therapeutic targets 68.

### Histological Markers

Histological markers, analyzed directly in CRC tumor tissues, are essential for diagnosis, prognosis, and personalized treatment. Factors such as tumor differentiation grade, submucosal invasion depth (>1 mm), lymph node involvement, perineural invasion, chronic inflammation, tumor budding, high-grade histology, preexisting adenomas, rectal localization, tumor margin, and endoscopic resection margins have been identified as the most relevant predictors of recurrence 69.

#### Cytokeratins

Through immunohistochemical techniques, cytokeratin analysis enables pathologists to determine the epithelial origin of tumors. Cytokeratin 20 (CK20), a type I cytokeratin, is primarily expressed in epithelial cells of the gastrointestinal and urinary tracts as well as in other specialized epithelial structures. Its presence is particularly useful in identifying gastrointestinal-origin tumors. Combined cytokeratin studies are frequently used to diagnose tumors or metastatic lesions of unknown origin. For instance, negativity for CK7 and positivity for CK20 are characteristic of colorectal tumors 70.

#### Proliferation Markers

In colon cancer, proliferation markers such as **Ki-67**, **p16**, and **p21** positively or negatively regulate the cell cycle and are critical for assessing both proliferative activity and tumor prognosis.

- Ki-67: A nuclear protein expressed in proliferating cells during the active phases of the cell cycle (G1, S, G2, and M). A high Ki-67 index typically indicates a more aggressive tumor with a higher growth rate and lower differentiation, correlating with poorer prognosis 71.
- p16: A tumor suppressor protein that regulates the cell cycle by inhibiting cyclin-dependent kinases (CDKs), preventing progression from G1 to S phase. In colon cancer, loss or reduced expression of p16 is associated with uncontrolled proliferation, promoting tumor progression 71.
- p21: A cell cycle inhibitor regulated by p53, which halts the cell cycle in G1 or G2 phases to allow DNA repair or induce apoptosis. In colon cancer, increased p21 expression may reflect a protective response to cellular damage, whereas its loss is linked to uncontrolled proliferation and higher tumor aggressiveness 72.

Histological, genetic, and epigenetic markers play a pivotal role in the diagnosis, prognosis, and treatment of colorectal cancer (CRC). These biomarkers provide valuable insights into tumor biology, guiding risk stratification and personalized treatment strategies. Genetic alterations, such as mutations in APC, KRAS, NRAS, BRAF, and PI3K, alongside MSI and PTEN disruptions, highlight the complexity of CRC pathways and their impact on disease progression and therapy resistance. Similarly, epigenetic modifications, particularly aberrant methylation patterns, influence tumor suppressor gene activity and therapeutic responses.

Histological markers, including cytokeratins and proliferation-related proteins like Ki-67, p16, and p21, offer crucial prognostic information and help define tumor aggressiveness. The integration of these markers into routine clinical practice enables precise patient stratification, more effective therapeutic interventions, and improved outcomes. Continued research into these biomarkers will further enhance CRC management, offering opportunities for innovative targeted therapies and improved survival rates.

## MicroRNAs

MicroRNAs (miRNAs) are small non-coding RNA molecules, typically 18-25 nucleotides long, that regulate gene expression at the post-transcriptional level. They function by binding specifically to complementary sequences in the messenger RNA (mRNA) of target genes, leading to mRNA degradation or translational inhibition. This regulatory process allows miRNAs to influence various cellular pathways, including proliferation, apoptosis, differentiation, and invasion, which are fundamental processes in tumor progression. In colorectal cancer (CRC), miRNAs play a significant role in both oncogenesis and tumor progression, making them useful biomarkers for diagnosis, prognosis, prediction of treatment response, or therapeutic applications 73. The clinical utility of the diverse microRNA are summarized in Table 3.

Bandrés *et al.* used real-time polymerase chain reaction (PCR) to analyze the expression of 156 mature miRNAs in 16 CRC cell lines and 12 non-tumoral colonic tissue samples. They identified 22 overexpressed and 22 underexpressed miRNAs across all CRC cell lines, grouping the lines based on the presence of mutations in the KRAS and BRAF genes. miRNAs such as miR-9, miR-9*, miR-95, miR-148a, miR-190, and miR-372 were overexpressed in KRAS-mutant lines, while BRAF-mutant lines exhibited lower levels of these miRNAs. Additionally, some miRNAs showed alterations linked to clinical features, including miR-145, miR-31, miR-96, miR-133b, miR-135b, and miR-183, which were consistently dysregulated in CRC. Notably, miR-31 levels correlated with more advanced cancer stages, suggesting its involvement in tumor progression and increased aggressiveness of CRC 74.

In terms of therapeutic applications, a Japanese study 75 evaluated the role of the let-7 miRNA family in CRC. Let-7 acts as a negative regulator of several oncogenes, including RAS, which is involved in signaling pathways and tumor growth. It also influences cell cycle progression and promotes apoptosis, key processes for controlling cellular growth. Akao *et al.* demonstrated that introducing the precursor of let-7a-1, located on chromosome 9q22.3, into human colon cancer cells resulted in significant suppression of cellular growth. They also observed reduced levels of RAS and c-MYC proteins, while the levels of their mRNAs remained unchanged. These findings suggest that let-7 plays a role in CRC cell growth and may provide a foundation for developing novel anticancer agents.

Scientific studies aim to clarify the role of specific molecules in the prognosis of various diseases. A notable example is the study conducted by Ma X. *et al.*, which investigated the association between the expression of the microRNA miR-199b and clinicopathological characteristics, as well as its impact on the prognosis of colorectal cancer (CRC) patients. The analysis included tumor and adjacent normal tissue samples from 202 treated patients. Results showed that miR-199b expression was significantly lower in tumor tissues compared to adjacent normal tissues. However, among tumor tissues, miR-199b expression varied based on the presence or absence of lymph node metastases. Notably, tissues with metastases exhibited higher miR-199b expression compared to those without metastases. Furthermore, an increasing trend in miR-199b expression was observed with advancing TNM stages of cancer. Regarding prognosis, patients with high miR-199b expression had a significantly lower five-year survival rate compared to those with low expression. The ROC curve indicated a cutoff value for miR-199b of -7.965, with an area under the curve of 0.578 (95% CI: 0.468-0.688), suggesting its potential utility as a prognostic marker 76.

Approximately 15% of colorectal carcinomas develop through the microsatellite instability (MSI) pathway, associated with mutations in genes involved in DNA mismatch repair. MSI tumors exhibit distinct pathological features and generally have a better clinical prognosis compared to microsatellite stable (MSS) tumors. A study analyzing miRNA and mRNA expression profiles in CRC with and without MSI employed techniques such as real-time PCR and Northern blot. Results revealed that miRNAs such as miR-25 and miR-92, members of the miR-17-92 family, were overexpressed in MSS samples. This suggests that these miRNAs might act as oncogenes, contributing to the more aggressive clinical behavior of MSS cancers compared to MSI cancers. These molecular expression findings could enhance the biomolecular characterization and classification of CRC, providing valuable information for understanding and managing the disease 77.

In conclusion, microRNAs hold significant potential as biomarkers in the molecular therapy of colorectal cancer (CRC). Published studies aim to integrate multiple microRNA biomarkers to optimize the diagnosis and treatment of these tumors. However, their clinical applicability faces several notable limitations that must be addressed.

One major challenge is the heterogeneity in analytical methods, as studies utilize diverse techniques such as reverse transcription quantitative polymerase chain reaction (RT-qPCR) and next-generation sequencing (NGS), making standardization and result comparison difficult 78, 79. Additionally, many studies involve small patient cohorts, reducing statistical power and increasing the risk of selection bias. Moreover, microRNA expression can vary significantly between individuals due to factors such as overall health, medication use, diet, and the tumor microenvironment, further complicating the analysis 80.

While certain microRNAs, such as miR-199b, show promise as prognostic biomarkers, their predictive values remain low. This is partly due to a lack of consensus on cutoff points and the need for validation through multicenter clinical trials with independent cohorts 81.

## Use of CTCs in Locoregional Disease to Prevent Recurrence

**Figure 2:**
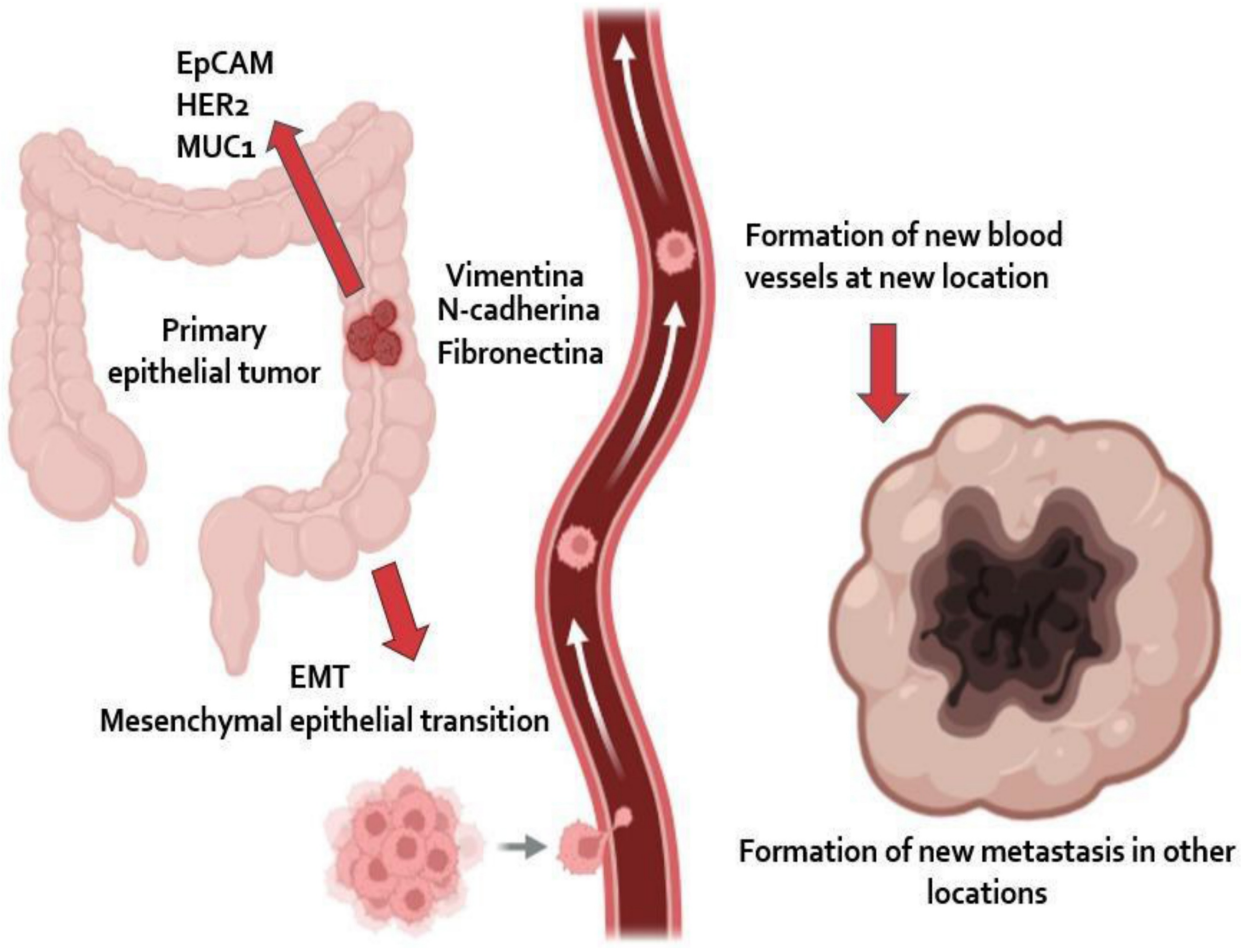
** Tumor Microenvironment and Circulating Tumor Cells (CTCs).** The image shows how a primary epithelial tumor expresses markers such as EpCAM, HER2, and MUC1, but undergoes epithelial-mesenchymal transition (EMT), acquiring migratory properties through the expression of Vimentin, N-cadherin, and Fibronectin. This enables CTCs to enter the bloodstream and travel to other organs, where they induce angiogenesis and form new metastases.

Circulating tumor cells (CTCs) are epithelial cancer cells primarily originating from solid epithelial tumors, such as those in the breast, prostate, colon, and lung. These nucleated cells express epithelial cell adhesion molecules (EpCAM) and/or cytokeratins (CK) in their cytoplasm but lack the common leukocyte antigen CD45. Unlike tumor cells confined to the primary site, CTCs are released into the circulatory system from the primary tumor or established metastases. After undergoing an epithelial-mesenchymal transition (EMT), they acquire stem cell-like characteristics, enabling them to migrate, invade blood vessels, and form new metastatic sites. As such, they are considered key contributors to *in vivo* tumor metastasis and provide molecular and biological insights into the tumor as a whole. Figure 2 illustrates how CTCs detach from the primary tumor, enter the bloodstream, and contribute to metastasis in other locations. By analyzing single cells, CTCs directly reflect changes occurring at various stages of tumor development 82. The clinical utility of the CTC are summarized in Table 4.

Several studies have demonstrated that a higher number of CTCs in diagnosed patients often correlates with greater disease aggressiveness, reduced survival, and an elevated risk of recurrence following treatment. This has led to their potential use in risk stratification, classifying patients according to their likelihood of recurrence or disease progression, which is particularly valuable in early or locoregional cancer stages 83, 84. Their presence is associated with poorer prognosis and a high likelihood of metastatic progression. Some studies have also reported the circulation of CTCs in bodily fluids before metastasis occurs, even during the early stages of the disease 85, 86.

By studying the genetic and molecular characteristics of CTCs, researchers can gain insights into specific cancer mutations or markers that may help predict patient response to certain treatments. This is especially critical in cases where the tumor has developed resistance to conventional therapies, as the molecular profile of CTCs can guide clinicians toward more effective and personalized therapeutic strategies 87.

The molecular characterization of circulating tumor cells (CTCs) offers a non-invasive approach to analyzing the genotypic and phenotypic features of tumors. These cells can be easily obtained from peripheral blood samples, although their presence in the bloodstream is extremely rare, with each CTC surrounded by 10⁶-10⁷ mononuclear leukocytes. Therefore, isolating CTCs from other blood cells is crucial for their study.

There are two main methods for isolating CTCs: label-dependent methods, which use specific cell surface markers, and label-independent methods, which rely on the physical or biological properties of tumor cells.

Label-dependent methods are based on the detection of specific molecules, such as epithelial cell adhesion molecule (EpCAM), which is present on most epithelial-origin cells but absent on blood cells. The **CellSearch® system** is a widely used commercial platform that captures CTCs via EpCAM. These cells are subsequently confirmed as negative for the hematopoietic marker CD45 and positive for cytokeratins. In addition to EpCAM, other markers, such as HER2 receptor, mucin 1 (MUC1), and cytokeratins, are also used for CTC characterization.

On the other hand, label-independent methods leverage the physical properties of CTCs, such as their density or size, to separate them from blood cells. Advanced technologies, including microfluidics and density gradients, have shown great promise in recognizing and isolating these cells. These techniques eliminate the reliance on specific markers, broadening the scope of study to include a greater diversity of tumor types 88.

Several studies support the use of circulating tumor cells (CTCs) in locally advanced colorectal cancer (CRC) to predict recurrence, guide adjuvant treatments, and optimize long-term surveillance. In a meta-analysis conducted by Lu *et al.*, the detection of CTCs in CRC patients was associated with shorter progression-free survival and an increased risk of recurrence, even in early stages. This relationship was observed even among patients traditionally classified as low-risk, suggesting that CTC analysis can identify individuals who may benefit from adjuvant chemotherapy after surgery. The meta-analysis consolidated data from multiple studies, reinforcing the prognostic value of CTCs in CRC, particularly in the locoregional context 89.

In the postoperative setting, a meta-analysis assessed the utility of CTCs for monitoring recurrence and metastasis after surgery. The results indicated that the presence of CTCs following surgical procedures was strongly associated with a higher risk of relapse and metastasis, underscoring their potential as predictive biomarkers in patients with early and localized CRC 90. Sotelo MJ *et al.* supported this evidence through a multicenter trial investigating the detection of CTCs in stage I-III CRC patients after surgery. The study revealed that patients with detectable postoperative CTCs faced a significantly higher risk of recurrence compared to those without detectable CTCs. This finding highlights the role of CTCs in identifying high-risk patients who could benefit from adjuvant treatments, even in cases where additional therapy might not traditionally be recommended 91.

Further supporting this, Rothé F. *et al.* demonstrated the relevance of CTCs using the CellSearch® system. Their research showed that detecting CTCs in postoperative patients allowed for more accurate identification of those at risk of relapse, facilitating personalized treatment plans and long-term surveillance strategies. The study emphasized the value of CTCs for risk stratification, particularly in patients considered low-risk based on traditional criteria 92.

The use of CTCs as real-time monitoring markers has also been validated by numerous clinical studies. A systematic review by Tan *et al.* highlighted that a reduction or disappearance of CTCs following chemotherapy correlates with improved therapeutic responses in CRC. This finding enables treatment efficacy to be assessed without solely relying on radiological imaging. Conversely, the persistence or increase of CTCs may indicate residual disease, justifying adjustments in management, such as therapy intensification or switching treatments, thereby optimizing outcomes in locoregional CRC 93.

Molecular characterization studies of circulating tumor cells (CTCs) have demonstrated their ability to provide critical information about tumor mutations and characteristics, which is essential for predicting resistance to certain treatments. By analyzing CTCs at the genetic level, specific alterations such as mutations in KRAS, BRAF, or PIK3CA can be identified, each carrying significant clinical implications. For instance, KRAS/NRAS mutations in metastatic colorectal cancer (mCRC) are associated with resistance to EGFR-targeted therapies, such as cetuximab and panitumumab. Similarly, BRAF mutations, particularly BRAF V600E, are linked to poorer prognosis and can guide the use of combination therapeutic strategies, such as BRAF inhibitors alongside other targeted agents, optimizing clinical management for these patients 94.

**Figure 3:**
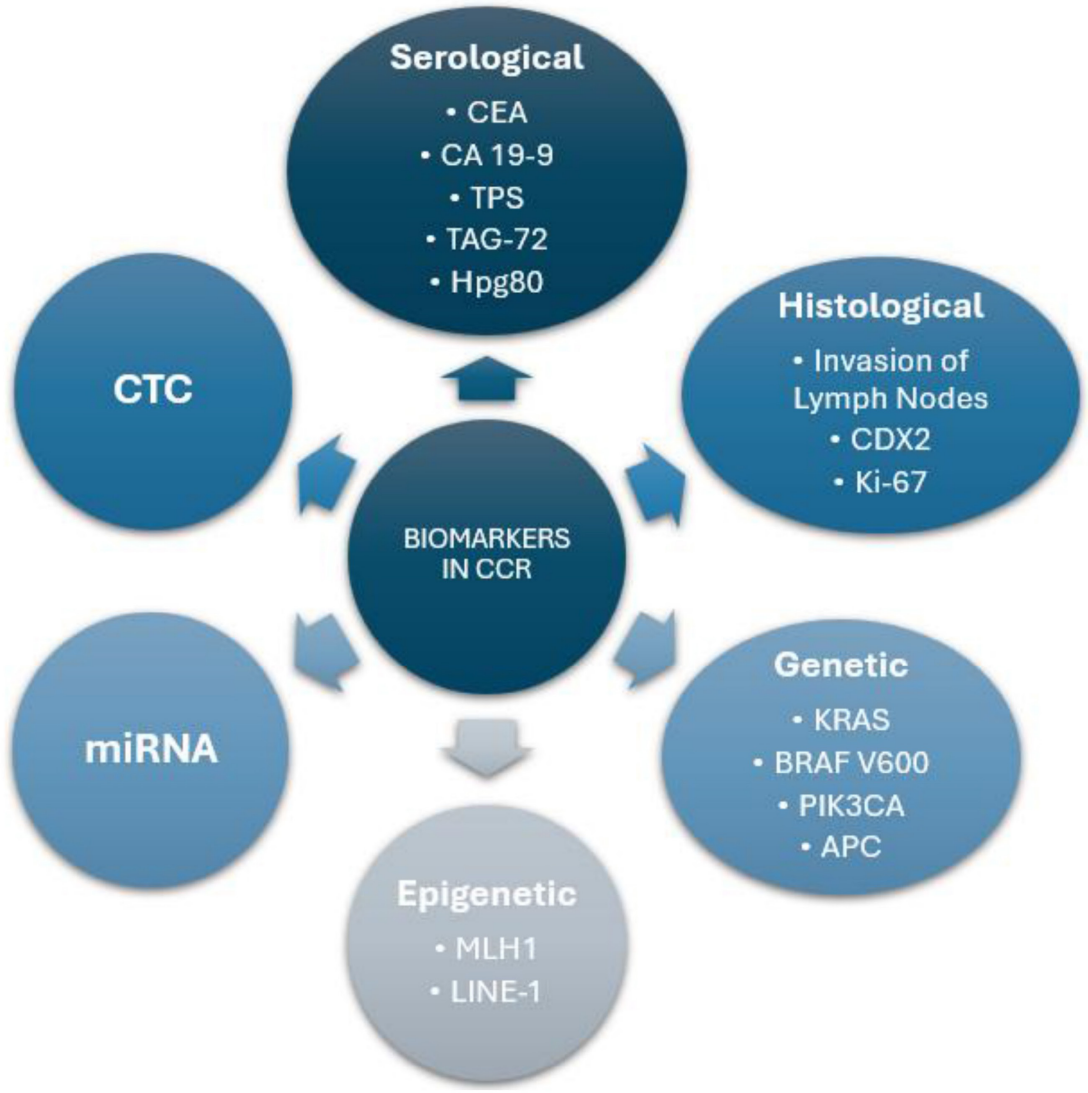
** Summary of Biomarker Categories in Colorectal Cancer.** This diagram represents a general classification of the biomarkers used in colorectal cancer, differentiating them by type and providing key examples within each category.

This approach represents a significant step forward in precision medicine, enabling treatments to be tailored to the unique biological characteristics of each patient's tumor. The dynamic ability of CTCs to reflect the molecular evolution of cancer makes them an ideal tool for real-time monitoring, facilitating therapy adjustments in response to emerging resistances or changes in tumor profiles. A summary of the biomarker categories in colorectal cancer is shown in Figure 3.

## Conclusions

This review highlights the pivotal role of advanced biomarkers, including histological, genetic, epigenetic, microRNA, and circulating tumor cell (CTC) analyses, in the management of colorectal cancer (CRC). These biomarkers not only enhance our understanding of CRC biology but also improve diagnostic accuracy, prognostic stratification, and treatment personalization.

Histological markers remain essential in assessing tumor aggressiveness, recurrence risk, and therapy responses. Genetic biomarkers, such as APC, KRAS, NRAS, BRAF, PIK3CA, PTEN, TP53, and NDST4, reveal critical molecular pathways involved in CRC progression and provide actionable targets for precision therapies. Epigenetic modifications, particularly DNA methylation patterns, offer additional layers of insight, enabling refined classification of CRC phenotypes and identifying novel therapeutic opportunities. The integration of microRNAs as biomarkers demonstrates their potential in CRC diagnosis, prognosis, and therapeutic applications, despite challenges in clinical standardization. Lastly, the study of CTCs has emerged as a transformative tool, offering real-time, non-invasive monitoring of tumor dynamics and identifying molecular changes that drive resistance or recurrence.

Although these advances bring us closer to fully realizing precision medicine in CRC, several challenges remain. Variability in analytical methodologies, limited cohort sizes, and inter-patient heterogeneity pose obstacles to the standardization and clinical application of these biomarkers. The need for multicenter validation studies and harmonization of techniques is critical to overcoming these limitations.

In conclusion, the integration of diverse biomarkers into clinical workflows has the potential to revolutionize CRC management by enabling early detection, improving prognostic accuracy, guiding therapy selection, and optimizing long-term surveillance. Ongoing research and collaboration across disciplines will be essential to translate these advances into tangible benefits for patients, ushering in a new era of personalized care in colorectal cancer.

## Figures and Tables

**Table 1:** Serological Biomarkers.

First author/s, year	Biomarker	Marker type	Diagnostic or prognostic utility	Refs.
Duffy MJ *et al.*, 2003 - Yuan SQ *et al.*, 2008.	Carcinoembryonic Antigen (CEA)	Serological	Key marker for post-surgical surveillance; predicts recurrence and liver metastasis	20, 21
Nakayama T *et al.*, 1997 - Filella X *et al.*, 1994	Carbohydrate Antigen 19-9 (CA 19-9)	Serological	Associated with poor prognosis; less sensitive for early recurrence detection	24, 25
Mishaeli M *et al.*, 1998 - Kucera R *et al.*, 2016	Tissue Polypeptide Specific Antigen (TPS)	Serological	Reflects tumor proliferation; elevated in advanced cases	26, 27
Guadagni F *et al.*, 1993	Tumor-Associated Glycoprotein 72 (TAG-72)	Serological	Complementary marker to CEA for monitoring CRC	29
You B *et al.*, 2022 - You B *et al.*, 2022	hPG80 (circulating progastrin)	Serological	Potential marker for early detection and disease monitoring	31, 32

The table highlights key serological biomarkers used in colorectal cancer management, emphasizing their diagnostic and prognostic roles. Biomarkers such as CEA are critical for detecting recurrences and metastases, while CA 19-9 is linked to poor prognosis. Emerging markers like hPG80 show promise for early detection and disease monitoring.

**Table 2:** Comprehensive Histological, Genetic, and Epigenetic Biomarkers

First author/s, year	Biomarker	Marker type	Diagnostic or prognostic utility	Refs.
Popat S *et al.*, 2005	Microsatellite Instability (MSI)	Genetic	Indicates better prognosis and resistance to monotherapy with fluoropyrimidines; associated with immune infiltration and better survival outcomes.	41
Zhu G *et al.*, 2021 - Yamashita S *et al.*, 2028	KRAS/NRAS	Genetic	Associated with poor prognosis; predicts resistance to anti-EGFR therapies; frequent in metastatic colorectal cancer.	45, 47
Tabernero J *et al.*, 2021	BRAF V600E	Genetic	Linked to high-grade tumors with poor prognosis; prevalent in right-sided tumors and older patients.	51
Tran B *et al.*, 2011	Invasion of Lymph Nodes	Histological	Predicts recurrence risk and poor outcomes; reflects aggressive local invasion.	69
Liao X *et al.*, 2012	PIK3CA	Genetic	Associated with poor prognosis; mutations in exons 9 and 20 predict worse survival; aspirin use may improve outcomes.	53
Fakih M *et al.*, 2024	KRAS G12C	Genetic	Predicts response to therapies targeting KRAS mutations; improved progression-free survival with targeted therapy combinations.	46
Zhang L *et al.*, 2017 - Liang J *et al.*, 2013	APC Mutation	Genetic	Essential in tumor progression; linked to instability in WNT signaling pathway; predictive of worse outcomes in advanced disease.	38, 39
Wright M *et al.*, 2017	Hypermethylation (MLH1)	Epigenetic	Correlates with microsatellite instability; better prognosis in tumors with hypermethylation of MLH1 promoter.	62
Hur K *et al.*, 2014	Hypomethylation (LINE-1)	Epigenetic	Linked to aggressive tumor behavior and poor prognosis; found in advanced colorectal cancer.	66
Ilie-Petrov *et al.*, 2023	CDX2	Histological/Epigenetic	Expression correlates with better survival; loss linked to tumor progression and worse outcomes.	68

The table on histological markers highlights their critical role in colorectal cancer (CRC) diagnosis, prognosis, and treatment planning. It includes key factors such as tumor differentiation grade, depth of submucosal invasion, lymph node involvement, and specific molecular markers like cytokeratins (e.g., CK20). Additionally, markers like Ki-67, p16, and p21 provide insights into tumor proliferation and aggressiveness, guiding risk stratification and therapeutic strategies.

**Table 3:** MicroRNA Biomarkers

First author/s, year	Biomarker	Marker type	Diagnostic or prognostic utility	(Refs.)
Bandrés *et al.*, 2006	miR-31	MicroRNA	Correlates with advanced stages of colorectal cancer; indicates tumor progression and aggressiveness.	74
Akao *et al.*, 2007	let-7	MicroRNA	Regulates oncogenes like RAS; shows potential as a therapeutic agent to suppress tumor growth.	75
Ma X *et al.*, 2020	miR-199b	MicroRNA	Lower expression in tumors correlates with worse survival; high levels linked to advanced stages.	76
Hur K *et al.*, 2014	miR-17-92 (miR-25, miR-92)	MicroRNA	Overexpression linked to aggressive clinical behavior in MSS tumors.	77

The microRNA table details their regulatory role in CRC biology, including tumor progression, metastasis, and therapy resistance. It highlights specific miRNAs, such as miR-31, associated with advanced cancer stages and aggressiveness, and let-7, which regulates oncogenes like RAS. These markers serve as potential diagnostic and prognostic tools and provide therapeutic opportunities for personalized treatments.

**Table 4:** Biomarkers of Circulating Tumor Cells

First author/s, year	Biomarker	Marker type	Diagnostic or prognostic utility	(Refs.)
Lu *et al.*, 2020	Circulating Tumor Cells (CTCs)	Cellular Biomarker	Associated with worse progression-free survival and higher recurrence risk in early stages.	(89)
Sotelo MJ *et al.*, 2021	Post-surgical CTCs	Cellular Biomarker	Detection after surgery predicts recurrence and metastasis, guiding adjuvant therapy.	(91)
Tan *et al.*, 2022	CTCs reduction	Cellular Biomarker	Reduction after chemotherapy correlates with better therapeutic response.	(93)
Rothé F *et al.*, 2021	Molecularly characterized CTCs	Cellular Biomarker	Provide insights into tumor genotypes for personalized treatment strategies.	(92)
Pantel K *et al.*, 2019	CTCs with KRAS/BRAF mutations	Cellular Biomarker	Predict resistance to EGFR-targeted therapies and guide treatment adjustments.	(94)

This table emphasizes their utility as non-invasive biomarkers for monitoring CRC. It covers their role in predicting recurrence, assessing treatment response, and guiding adjuvant therapy. Methods for isolation and analysis, such as EpCAM-based detection and label-independent techniques, are detailed, showcasing how CTCs provide real-time insights into tumor evolution and resistance mechanisms.
